# Systemic immunological profile of children with B-cell acute lymphoblastic leukemia: performance of cell populations and soluble mediators as serum biomarkers

**DOI:** 10.3389/fonc.2023.1290505

**Published:** 2023-12-01

**Authors:** Maria Perpétuo Socorro Sampaio Carvalho, Fábio Magalhães-Gama, Bruna Pires Loiola, Juliana Costa Ferreira Neves, Nilberto Dias Araújo, Flavio Souza Silva, Claudio Lucas Santos Catão, Eliana Brasil Alves, João Paulo Diniz Pimentel, Maria Nazaré Saunier Barbosa, Nelson Abrahim Fraiji, Andréa Teixeira-Carvalho, Olindo Assis Martins-Filho, Allyson Guimarães Costa, Adriana Malheiro

**Affiliations:** ^1^ Diretoria de Ensino e Pesquisa, Fundação Hospitalar de Hematologia e Hemoterapia do Amazonas (HEMOAM), Manaus, Brazil; ^2^ Programa de Pós-Graduação em Ciências Aplicadas à Hematologia, Universidade do Estado do Amazonas (UEA), Manaus, Brazil; ^3^ Programa de Pós-Graduação em Ciências da Saúde, Instituto René Rachou - Fundação Oswaldo Cruz (FIOCRUZ) Minas, Belo Horizonte, Brazil; ^4^ Programa de Pós-Graduação em Medicina Tropical, Universidade do Estado do Amazonas (UEA), Manaus, Brazil; ^5^ Programa de Pós-Graduação em Imunologia Básica e Aplicada, Instituto de Ciências Biológicas, Universidade Federal do Amazonas (UFAM), Manaus, Brazil; ^6^ Hospital Universitário Getúlio Vargas, Universidade Federal do Amazonas (UFAM), Manaus, Brazil; ^7^ Escola de Enfermagem de Manaus, Universidade Federal do Amazonas (UFAM), Manaus, Brazil

**Keywords:** childhood leukemia, cellular immunity, chemokines, cytokines, induction therapy, biomarkers, liquid biopsy

## Abstract

**Background:**

Children with B-cell acute lymphoblastic leukemia (B-ALL) have an immune imbalance that is marked by remodeling of the hematopoietic compartment, with effects on peripheral blood (PB). Although the bone marrow (BM) is the main maintenance site of malignancy, the frequency with which immune cells and molecules can be monitored is limited, thus the identification of biomarkers in PB becomes an alternative for monitoring the evolution of the disease.

**Methods:**

Here, we characterize the systemic immunological profile in children undergoing treatment for B-ALL, and evaluate the performance of cell populations, chemokines and cytokines as potential biomarkers during clinical follow-up. For this purpose, PB samples from 20 patients with B-ALL were collected on diagnosis (D0) and during induction therapy (days 8, 15 and 35). In addition, samples from 28 children were used as a control group (CG). The cellular profile (NK and NKT-cells, Treg, CD3^+^ T, CD4^+^ T and CD8^+^ T cells) and soluble immunological mediators (CXCL8, CCL2, CXCL9, CCL5, CXCL10, IL-6, TNF, IFN-γ, IL-17A, IL- 4, IL-10 and IL-2) were evaluated via flow cytometry immunophenotyping and cytometric bead array assay.

**Results:**

On D0, B-ALL patients showed reduction in the frequency of cell populations, except for CD4^+^ T and CD8^+^ T cells, which together with CCL2, CXCL9, CXCL10, IL-6 and IL-10 were elevated in relation to the patients of the CG. On D8 and D15, the patients presented a transition in the immunological profile. While, on D35, they already presented an opposite profile to D0, with an increase in NKT, CD3^+^ T, CD4^+^ T and Treg cells, along with CCL5, and a decrease in the levels of CXCL9, CXCL10 and IL-10, thus demonstrating that B-ALL patients present a complex and dynamic immune network during induction therapy. Furthermore, we identified that many immunological mediators could be used to classify the therapeutic response based on currently used parameters.

**Conclusion:**

Finally, it is noted that the systemic immunological profile after remission induction still differs significantly when compared to the GC and that multiple immunological mediators performed well as serum biomarkers.

## 1 Introduction

B-cell acute lymphoblastic leukemia (B-ALL) is the most common pediatric cancer in the world and is characterized by an abnormal proliferation of malignant B-cell precursors in the bone marrow (BM), which results in their release into peripheral blood (PB) and extramedullary tissues (1, 2). Recently, with the introduction of intensive multi-agent chemotherapy regimens and therapy adapted to risk groups, the treatment of childhood B-ALL has significantly advanced, reaching 90% event-free survival (3, 4). However, a significant number of children have side effects and develop comorbidities induced by chemotherapy, with approximately 20% of patients with B-ALL suffering from relapses and showing an unsatisfactory therapeutic response (5–7).

Clinical, biological, and genetic characteristics have been reported to be predictors of the response to therapy and the likelihood of relapse in children with B-ALL (3, 8, 9). In addition, studies have shown that characteristics related to the immunological profile, based on the levels of cytokines and chemokines, in addition to the frequency and phenotype of cell populations, can also be used as potential prognostic biomarkers in children and adults (10–15). Furthermore, our studies have previously demonstrated, through characterization of the network of soluble immunological mediators in the bone marrow compartment, that particular mediators can provide valuable information about the stage of malignancy in relation to leukemic burden, risk group stratification, and detection of measurable residual disease (16, 17).

In fact, evidence indicates that children with B-ALL have an imbalance in the soluble immunological mediators, already at birth, which accompanies the different stages of development and progression of the disease (16, 18–20). Thus, after malignant transformation, as leukemic cells replace normal hematopoietic stem cells, there is a remodeling in the immunological landscape, which is marked by changes in cell phenotypes and changes in the network of immunological mediators. These events aim to support leukemogenesis, cell survival, self-renewal, immune evasion and therapeutic resistance by providing an immunosuppressive and permissive microenvironment for leukemic progression (21–24).

Although the bone marrow is the main site of control and maintenance of malignancy, the frequency with which components of the leukemic microenvironment can be monitored is limited, especially in children. These problems are associated with the discomfort and practical difficulties posed by collecting bone marrow samples. However, this difficulty can be overcome by identifying evaluation methods related to leukemic cells, immunity to cancer and normal hematopoiesis, at the systemic level, in peripheral blood (liquid biopsy), therefore allowing sequential monitoring of the disease’s evolution. Thus, we characterize here the systemic immunological profile in children with B-ALL undergoing remission induction therapy, and evaluate the performance of these immunological mediators as potential biomarkers. Our data demonstrate that these patients exhibit a complex and dynamic immune network during remission induction; many immunological mediators exhibited high performance for classifying therapeutic response and may be potential serum biomarkers.

## 2 Materials and methods

### 2.1 Study population and design

This study was carried out at Fundação Hospitalar de Hematologia e Hemoterapia do Amazonas (HEMOAM), which is the state referral center for diagnosis and treatment of hematological diseases and is located in Manaus, Amazonas state, Brazil. A total do 20 patients with newly diagnosed B-cell acute lymphoblastic leukemia (B-ALL), of either gender (14 males and 6 females, median age = 3 years; interquartile range [IQR] = 2-9) were enrolled. The diagnosis was performed according to the classification criteria and guidelines of the World Health Organization (25). In addition, 28 children without leukemia, of either gender (15 males and 13 females, median age = 7 years; IQR = 4-11) were included as a control group (CG). B-ALL patients underwent remission induction therapy, according to the protocol and guidelines found in the Brazilian Group for Treatment of Childhood Leukemia (version 2009) (GBTLI-LLA-2009). Remission induction therapy consists of an intensive chemotherapy stage of fundamental importance for the prognosis of patients, whose objective is to achieve complete clinical remission in four weeks. The treatment regimen includes the drugs prednisone, dexamethasone, vincristine, daunorubicin, L-asparaginase and MADIT (intrathecal methotrexate, cytarabine and dexamethasone) (26). The demographical and clinical features of the study population are summarized in **Table 1**.

**Table 1:** Demographical and clinical features of the study population.

Variables	CG (n=28)	B-ALL (n=20)
**Gender (n, male/female)**	15/13	14/6
**Age (median [IQR])**	7 [4-11]	3 [2-9]
**Age group**		
1 to <5	9 (65%)	13 (65%)
5 to <9	9 (5%)	1 (5%)
9 to <14	7 (15%)	3 (15%)
14 to <18	3 (15%)	3 (15%)
**Immunophenotyping**		
Common B-ALL (CD10^+^)	-	20 (100%)
**Treatment risk stratification on D0**		
Low Risk (LR)	-	13 (65%)
High Risk (HR)	-	7 (35%)
**Treatment risk re-stratification on D15**		
Low risk (LR)	-	9 (45%)
High risk (HR)	-	11 (55%)
**Measurable residual disease on D35**		
MRD^(-)^	-	9 (45%)
MRD^(+)^	-	11 (55%)

CG, control group; IQR, interquartile range; MRD, measurable residual disease.

### 2.2 Ethical approval and consent to participate

The study was submitted to and approved by the Ethical Committee at Fundação HEMOAM, under the protocol registration number #739.563/2014. The parents or legal guardians read and signed the informed consent form prior to inclusion of the pediatric subjects in the study. The study fulfills the principles of the Helsinki declaration and the 466/2012 resolution of the Brazilian National Health Council for research involving human participants.

### 2.3 Data collection and peripheral blood sampling

Demographic and clinical data were collected from medical records kept at the Medical & Statistical Service, as well as from the Hematology Laboratory records. Peripheral blood samples were obtained via venipuncture in EDTA vacuum tubes (BD Vacutainer EDTA K2) at four consecutive time points during induction therapy in accordance with the protocols and guidelines of the GBTLI-LLA-2009 (26), and denominated as: on diagnosis baseline (D0); day 8 and day 15 of induction therapy (D8 and D15, respectively); and at final of remission induction therapy (D35). In parallel, peripheral blood samples from children without leukemia were used as a reference value in the analyses. The children included in the study had not had any previous infections for at least four weeks and did not present any immunological changes in the leukocyte series. Patients and controls samples were subjected to centrifugation at 900 x g for 15 min at 4°C to obtain approximately 1 mL of plasma samples. Subsequently, the plasma was aliquoted and immediately stored at -80°C until processing for the quantification of soluble immunological mediators that occurred within 6 months after collection. Finally, the whole blood was used for immunophenotyping to evaluate cell populations.

### 2.4 Immunophenotypic characterization

The immunophenotypic characterization was performed using flow cytometry. The cells were obtained from an aliquot of 100 μL of peripheral blood and were incubated in the presence of the following fluorescent-labeled specific anti-human cell surface monoclonal antibodies: anti-CD3-PercP/CD16-FITC/CD56-PE to identify natural killer cells (NK) and natural killer T cells (NKT); anti-CD3-PercP/CD4-PE/CD8-FITC to identify CD3^+^ T cells, CD4^+^ T cells and CD8^+^ T cells; and anti-CD4-PE/CD25-FITC/FoxP3-APC to identify regulatory T cells (Treg). Following the incubation, the cells were treated with 2 mL of erythrocyte BD FACS™ Lysing Solution for 10 min at room temperature. After two centrifugation steps and two wash steps with PBS, the cells were resuspended in 300 μL PBS. For intracellular labeling of FoxP3, the cells were fixed with BD Cytofix™ Fixation Buffer and permeabilized with BD Phosflow™ Perm Buffer III. Centrifugation, washing, and incubation steps were carried out with anti-FoxP3-APC antibody for 20 minutes at room temperature and protected from light. At the end, the samples were acquired in the cytometer. The sample acquisition was performed in a flow cytometer (FACSCalibur, BD^®^ Biosciences, San Jose, CA, USA) at Fundação HEMOAM. A total of 10,000 or 100,000 (Treg cells) events were acquired for each sample in order to quantify cell populations. Lymphocyte subsets were quantified first by specific gating strategies, using the FlowJo software (version 9.4.1, TreeStar Inc. Ashland, OR, USA) as represented in **Supplementary Figure 1**. The results were expressed initially as percentage of positive cells within the lymphocyte gate. Furthermore, FlowJo software was used for the morphometric and immunophenotypic identification of the cells with the gates designed to select the target populations in graphs that combine morphological characteristics (size and complexity) with immunophenotypic characteristics through the fluorescence of the monoclonal antibodies used to identify the target cells.

### 2.5 Quantification of soluble immunological mediators

Soluble immunological mediators, including chemokines and cytokines (CXCL8, CCL2, CXCL9, CCL5, CXCL10, IL-6, TNF, IFN-γ, IL-17A, IL-4, IL-10 and IL-2) were measured from the peripheral blood plasma samples using cytometric bead array (CBA) (BD™ Human Chemokine and BD™ Human Cytokine Th1/Th2/Th17 kits, San Diego, CA, USA) assays, according to the manufacturer’s instructions. Samples were evaluated in a flow cytometer (FACSCantoII, BD^®^ Biosciences, San Jose, CA, USA) at Instituto Leônidas e Maria Deane (FIOCRUZ Amazônia). FCAP-Array software v3 (Soft Flow Inc., USA) was used for data analysis. Data were reported in picograms per milliliter (pg/mL) concentrations, according to the standard curves provided in the kit and mean fluorescence intensity (MFI).

### 2.6 Conventional data analysis and ROC curve

The comparative analysis between B-ALL patients and controls was carried out using Student’s t test. Multiple comparisons among the time points of induction therapy (D0, D8, D15 and D35) were performed using one-way ANOVA followed by the Tukey or Friedman tests followed by Dunn’s test; along with the paired t test or Wilcoxon matched-pairs signed-ranks test. In all cases, the Shapiro-Wilk test was used to verify the normality of the data and significance was considered when p was <0.05. The receiver-operating characteristic curves (ROC) and the area under the curve (AUC) were used as an indicator of global accuracy (27). Performance of the cell populations and the selected soluble immunological mediators (statistically significant - p<0.05) was calculated at a specific cut-off, and the AUC, sensitivity (Se), specificity (Sp), and likelihood ratio (LR) were considered as indicators of global accuracy. The GraphPad Prism Software v8.0.1 (San Diego, CA, USA) was used for statistical analysis and Biorender was used for graphical arts.

### 2.7 Ascendent curve of cell populations and soluble immunological mediators

The overall signature of the cell populations and soluble immunological mediators was determined according to Kerr et al. (2021) (17), by converting the original results of each variable expressed as a continuous variable into categorical data, using the global median values obtained for the whole data universe from all participants (B-ALL patients on different days of induction therapy and the controls) as the cut-off to segregate patients with low and high frequency of cell populations, and chemokines and cytokines levels. The following cut-off points were used: NK=4.18; NKT=1.96; CD3^+^ T=64.40; CD4^+^T=49.50; CD8^+^T=37.10; Treg=2.88; CXCL8 = 1058.64; CCL2 = 628.35; CXCL9 = 2001.07; CCL5 = 209502.20; CXCL10 = 4846.97; IL-6 = 216.79; TNF=83.44; IFN-γ=81.95; IL17A=81.95; IL-4 = 156.45; IL-10 = 123.67; and IL-2 = 137.08, expressed as a percentage for cell populations, and MFI for chemokines and cytokines. The assembling of the ascendant frequency of cell populations, and chemokines and cytokine levels of the CG generated the reference ascendent curves, which were applied to identify changes in the immunological profile of the B-ALL patients during induction therapy (D0, D8, D15 e D35). Relevant differences were considered when values were above the 50^th^ percentile of the study group. Color charts were used to summarize the proportion of subjects that presented decrease, unaltered or increase in immunological mediators in relation to the CG. A Venn diagram was designed (http://bioinformatics.psb.ugent.be/webtools/Venn/) and used to identify common and selective alterations at each time point.

### 2.8 Integrative network of cell populations and soluble immunological mediators

Correlation networks were assembled to evaluate the multiple associations among the immunological elements in B-ALL patients during induction therapy and in the controls. The association between the frequency of cell populations and levels of chemokines, cytokines and growth factors were determined by using the Pearson and Spearman correlation coefficient in GraphPad Prism, version 8.0.1 (GraphPad Software, San Diego, CA, USA), and statistical significance was considered only if p was <0.05. After performing the correlation analysis, a database was created using the Microsoft Excel program. Then, the significant correlations were compiled using the open source Cytoscape software, version 3.0.3 (National Institute of General Medical Sciences, Bethesda, MD, USA). Biological networks were constructed using circular layouts in which each molecule is represented by a globular node. The correlation indices (r) were used to categorize the correlation strength as negative (r <0), moderate (0.36≥ r ≤0.68), and strong (r >0.68), which were represented by connecting edges, as proposed by Taylor (1990) (28).

## 3 Results

### 3.1 B-ALL patients exhibit an increase in CD4^+^ T and CD8^+^ T cells along with a peculiar profile of soluble mediators marked by an increase in CXCL9, CXCL10, IL-6 and IL-10

**Figure 1:**
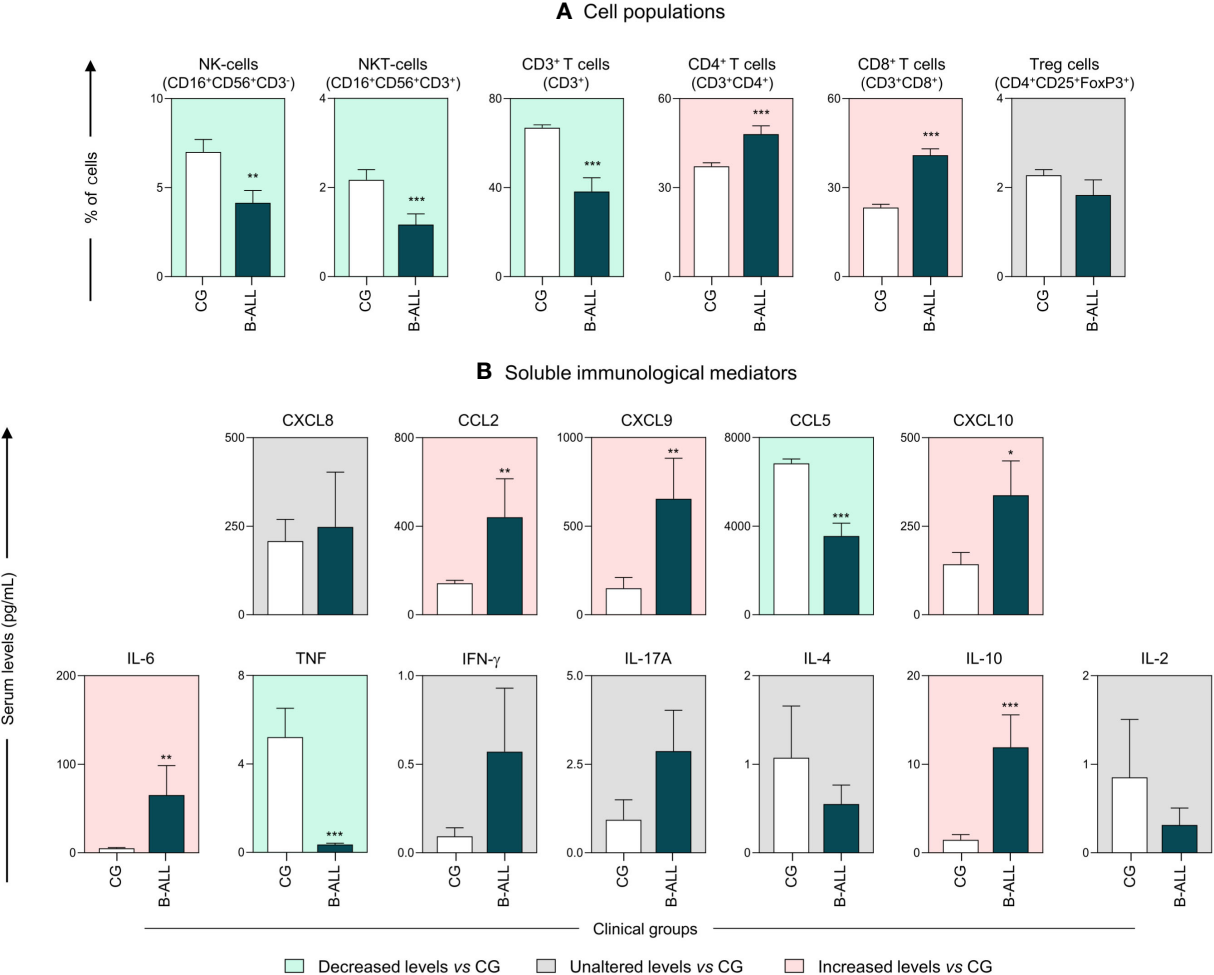
Characterization of the systemic immunological profile of pediatric patients diagnosed with B-ALL. The cell populations **(A)** and soluble immunological mediators **(B)** were measured at the time of diagnosis in B-ALL patients (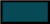) and in the control group (CG) (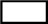). The behavior of immunological mediators in relation to the controls is highlighted using the background color in the following manner: decreased levels vs CG (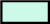); unaltered levels vs CG (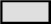); and increased levels vs CG (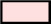). The immunophenotypic characterization was performed using flow cytometry, while chemokines and cytokines were quantified via CBA, as described in the Materials and Methods section. Data are reported as mean with standard error expressed in percentage of cells and in picograms per milliliter (pg/mL) concentration, respectively. Statistical analyses were performed using Student’s t test or the Mann-Whitney test and significant differences are highlighted by asterisks for p<0.001 (***) p<0.01 (**) or p<0.05 (*).

The characterization of the immunological profile of the B-ALL patients is presented in **Figure 1**. The data demonstrated that B-ALL patients displayed a lower frequency of NK-cells, NKT-cells, and CD3^+^ T. On the other hand, the number of CD4^+^ T and CD8^+^ T cells was higher increased (**Figure 1A**). In addition, the measurement of soluble immunological mediators demonstrated an increase in the chemokines CCL2, CXCL9 and CXCL10. Increased levels of IL-6 and IL-10 were also observed in B-ALL patients. In contrast, when compared to the CG, a decrease in levels of CCL5 and TNF was observed (**Figure 1B**).

### 3.2 B-ALL patients exhibit a clear change in the immunological profile after induction therapy

**Figure 2:**
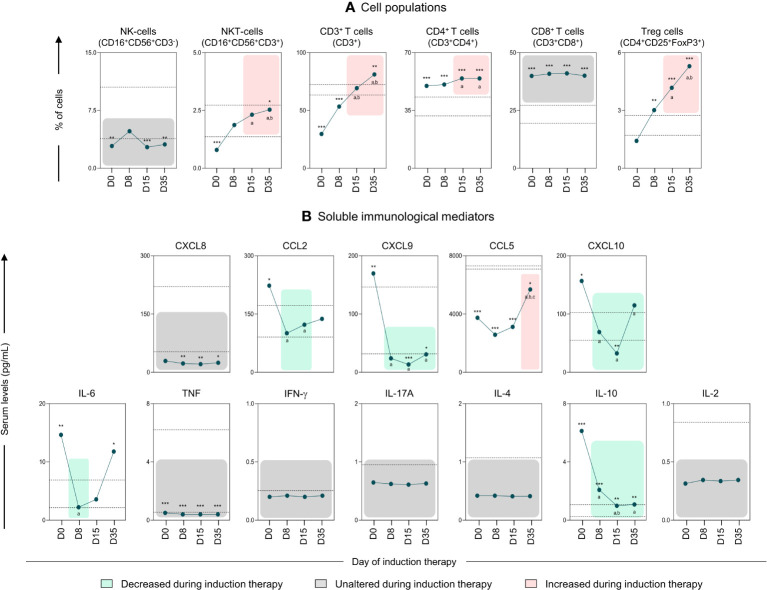
Kinetics of the systemic immunological mediators during induction therapy of pediatric patients with B-ALL. The cell populations **(A)** and soluble immunological mediators **(B)** were measured on D0, D8, D15, and D35 to assess the behavior of these immunological mediators during remission induction therapy. The behavior of immunological mediators during induction therapy were highlighted using the plotting area colors, in the following manner: decreased during induction therapy (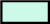); unaltered during induction therapy (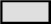); and increased during induction therapy (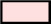). Data are reported as mean with standard error expressed in percentage of cells and in picograms per milliliter (pg/mL) concentration, respectively. Statistical analyses were performed using a paired t test or Wilcoxon matched-pairs signed-rank test for comparisons between D0, D8, D15 and D35, while Student’s t test or the Mann-Whitney test was used for comparison with the control group (CG). Significant differences (p<0.05) between the days of induction therapy are represented by the following letters: (a, b, c), which refer to the comparisons with D0, D8 and D15, respectively. Significant differences in relation to the CG are highlighted by asterisks for p<0.001 (***) p<0.01 (**) or p<0.05 (*).

Kinetic analysis of the cell populations, chemokines and cytokines was performed on D0, D8, D15, and D35 to assess the behavior of these immunological mediators during induction therapy, as shown in **Figure 2**. The data demonstrated that, compared to D0, on D35, B-ALL patients displayed an increase in most of the evaluated cell populations, including NKT-cells, T cells, CD4 T cells and Treg cells (**Figure 2A**). However, the dosage of soluble immunological mediators showed a significant decrease in levels of CXCL9, CXCL10 and IL-10 on D35, together with an increase in the levels of the chemokine CCL5. While CCL2 and IL-6 showed a decline on D8 and D15 (**Figure 2B**). In addition, compared to the CG, on D35, the B-ALL patients displayed a significant increase in the frequency of NKT-cells, CD3^+^ T, CD4^+^ T cells, CD8^+^ T cells, and Treg cells, along with an increase in IL-6 and IL-10 levels.

### 3.3 Overall signature of systemic immunological mediators highlighted distinct production profiles during induction therapy

**Figure 3:**
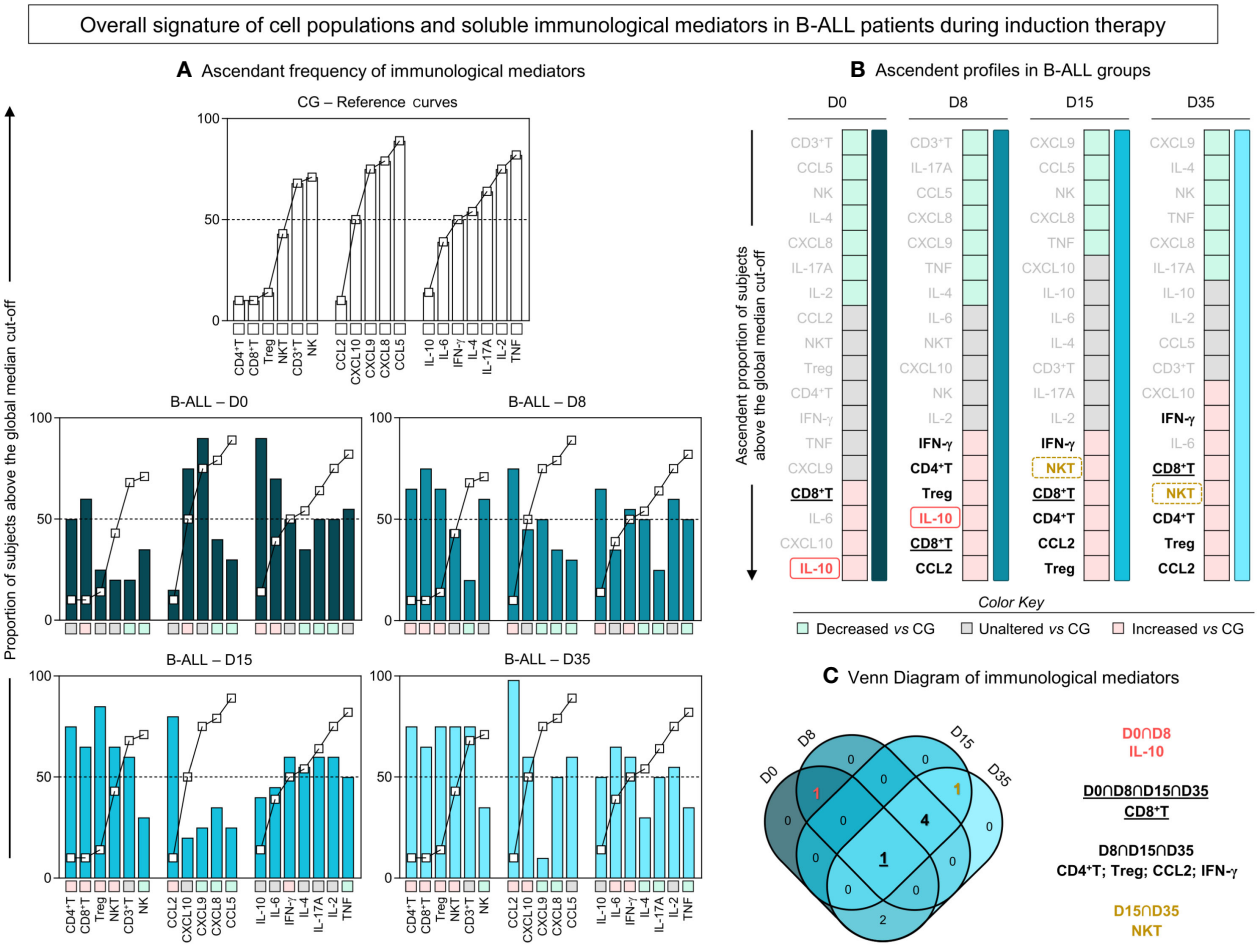
Overall signature of systemic immunological mediators during induction therapy of pediatric patients with B-ALL. **(A)** The overall signature of cell populations, chemokines and cytokines in B-ALL patients was assembled on D0, D8, D15, and D35. The ascendant frequency of the control group (CG) was used to generate the reference curves (-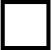-), which were applied to identify changes in the overall signature of B-ALL patients during induction therapy. Data, originally expressed as a percentage of cells and mean fluorescence intensity (MFI) were converted into categorical data using the global median values, which were used as a cut-off point to classify the study population as being a low or high producer of the parameters evaluated. The overall signatures were assembled in bar graphs using the 50^th^ percentile as the threshold (central line) to identify systemic immunological mediators with increased levels. **(B)** Color charts were used to summarize the ascending profile of immunological mediators on D0, D8, D15, and D35. The colors represent the behavior in relation to the CG, such as decreased levels vs CG (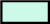), unaltered levels vs CG (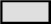), and increased levels vs CG (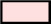). **(C)** Venn diagram analysis highlights common immunological mediators between days of induction therapy.

An additional analysis was carried out to further characterize the profile of immunological mediators in B-ALL patients, as presented in **Figure 3**. For this purpose, we calculated the global median for cell populations, chemokines and cytokines, and used these values as a cut-off point to classify the study population as being a low or high producer for the parameters evaluated. Then, according to the ascending frequency of production of the immunological mediators in the CG, reference curves were assembled and applied to identify changes during induction therapy in the immune signature of patients with B-ALL (**Figure 3A**). In addition, color charts were used to summarize the behavior of the immunological mediators (**Figure 3B**).

Based on this approach, the data demonstrated that on D0, there was a profile of lower production of immunological mediators, with high frequency/levels of CD8^+^ T, IL-6, CXCL10 and IL-10. On D8 and D15 of induction therapy, the B-ALL patients showed a similar production profile, with high frequency/levels of CD4^+^ T, CD8^+^ T, Treg, IFN-γ, CCL2 and, selectively, IL-10 and NKT, on D8 and D15, respectively. On D35, a distinct profile was observed in relation to D0, which was marked by increased production of immunological mediators, especially cell populations, with high frequency/levels of CXCL10, IFN-γ, IL-6, CD8^+^ T, NKT, CD4^+^ T, Treg and CCL2. Finally, a Venn diagram with the immunological mediators was used to identify common and selective alterations at each time point of induction therapy (**Figure 3C**). The analysis of the intersections showed that the high production of IL-10 occurs only at the beginning of the treatment, on D0 and D8; the frequency of NKT increases on D15 and D35; CD4^+^ T, Treg, CCL2 and IFN-γ increased on D8, D15 and D35; while the CD8^+^ T frequency was elevated throughout the induction therapy, from D0 to D35.

### 3.4 The integrative networks identify a robust participation of Treg cells, together with common correlations axis throughout induction therapy

**Figure 4:**
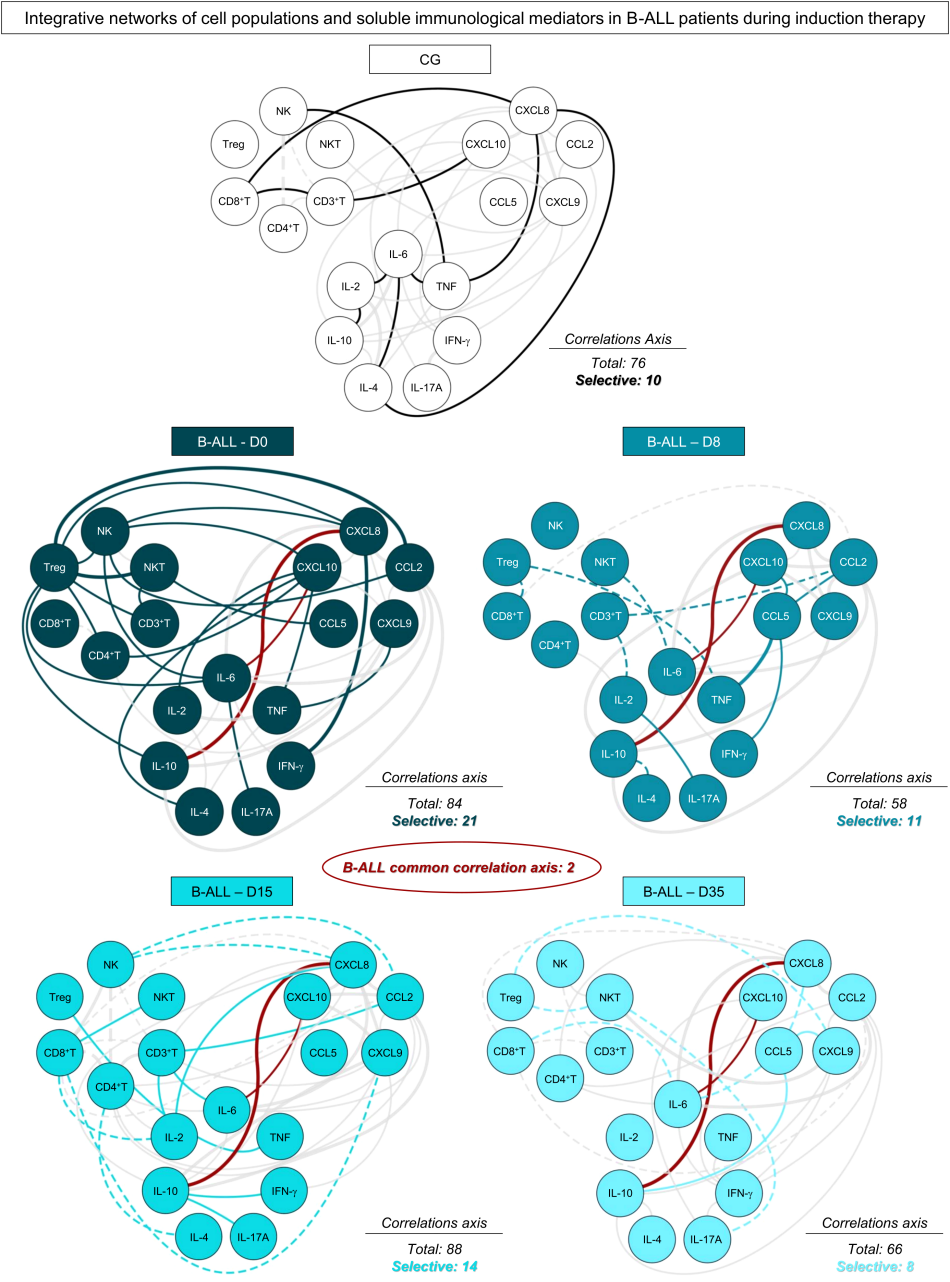
Networks of the systemic immunological mediators during induction therapy of pediatric patients with B-ALL. Integrative networks were assembled to identify the complex interactions among of cell populations, chemokines and cytokines during induction therapy. Colored nodes are used to identify the immunological mediators on D0 (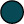), D8 (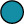), D15 (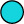) and D35 (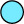) of B-ALL patients and in the control group (CG) (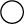). Correlation analysis was employed to construct integrative networks according to significant “r” scores at p<0.05 using Pearson and Spearman correlation tests. Connecting edges illustrate positive (continuous lines) and negative (dashed lines) correlations between pairs of attributes, according to the strength of correlation, as follows: weak (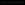); moderate (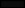); and strong (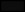), as described in the Materials and Methods section. Different colored lines are used to represent common connections between time points (gray lines) and selective connections (lines colored according to the respective time points).

In order to demonstrate the interactions between cell populations, chemokines and cytokines and during remission induction therapy, integrative networks were constructed, as shown in **Figure 4**. In addition, aiming to provide a more detailed view of the biological networks, the number of correlations and the selective axes of each time point were highlighted. The analysis of integrative networks revealed that, on D0, B-ALL patients selectively presented a network with a robust participation of Treg cells, and a greater number of connections between regulatory cytokines, related to the Th2 and Treg profile, in comparison to Th1 and Th17. On the other hand, the CG exhibited a discrete number of connections between cell populations, and were characterized by a mixed profile of T helper cytokines, and without interactions mediated by Treg cells.

During induction therapy, on D8, a clear decline in the number of correlations was observed, which was marked by the loss of interactions mediated by Treg cells, and the emergence of negative correlations. On D15, a significant increase was observed in the correlations, and this was marked by interactions between cell populations, with emphasis on CD4^+^ T and CD8^+^ T cells. Finally, on D35, B-ALL patients again showed a decrease in the number of correlations, and they evolved towards a pro-inflammatory profile, different from what was observed on D0, and was marked by increased connections between the cytokines IFN-γ and IL-17A, with a decrease in Treg and regulatory cytokines. Of note, two common correlations were observed throughout induction therapy, between CXCL10/IL-6 and CXCL8/IL-10.

### 3.5 Performance of systemic immunological mediators as prognostic biomarkers in B-ALL patients during induction therapy

**Figure 5:**
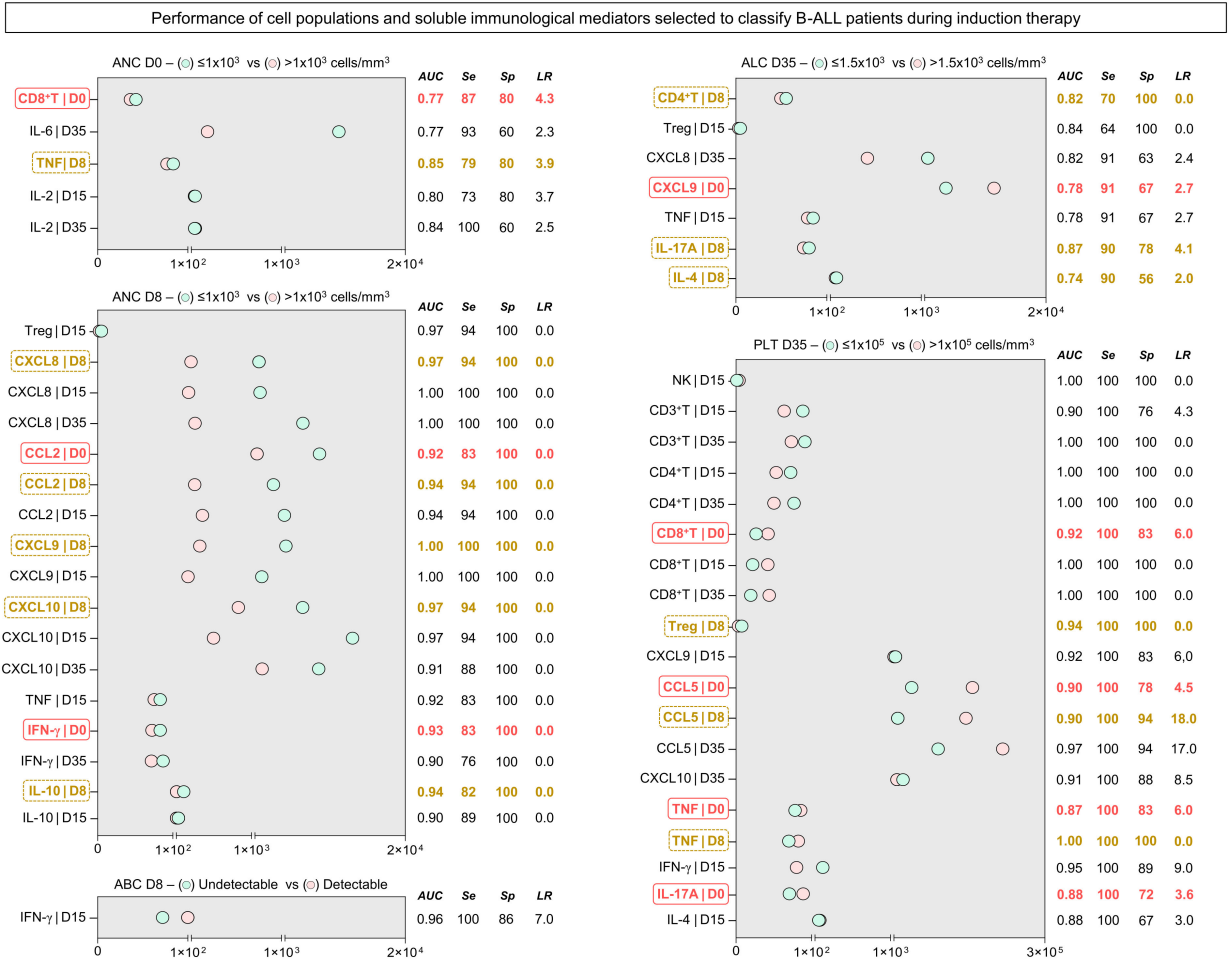
Performance of the selected systemic immunological mediators to classify patients with B-ALL during induction therapy. The performance of cell populations, chemokines and cytokines was evaluated by receiver-operating characteristic (ROC) curves to verify their global accuracy (estimated by the AUC values) according to classical laboratory parameters used for predicting the therapeutic response (ANC at D0 > 1x10^3^ cells/mm^3^; ANC at D8 > 1x10^3^ cells/mm^3^; Undetectable ABC at D8; ALC at D35 > 1.5x10^3^ cells/mm^3^; and PLT at D35 > 1x10^5^ cells/mm^3^). The selected immunological mediators (statistically significant - p<0.05) were calculated at specific cut-off and the area under the curve (AUC), sensitivity (Se), specificity (Sp), and likelihood ratio (LR) considered as an indicator of global accuracy. Early biomarkers (D0 and D8) are highlighted by solid red (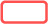) and dashed yellow rectangles (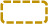), respectively. ANC, absolute neutrophil counts; ABC, absolute blast counts; ALC, absolute lymphocyte counts; PLT, platelet.

In order to transpose the findings for the immunological profile of B-ALL patients into clinically applicable biomarkers, translational analysis was carried out to determine the performance of cell populations and soluble immunological mediators measured in peripheral blood. Accordingly, ROC curves were constructed based on five laboratory parameters of classification of response to induction therapy (29–31) (**Supplementary Tables 1–5**). The performance (AUC, Se, Sp and LR) of the immunological mediators that had proven statistical significance is represented in **Figure 5**. Data analysis showed that several immunological mediators showed high performance in classifying patients based on the parameters used. For absolute neutrophil counts on D0, five potential biomarkers were identified, two of which are early biomarkers, CD8^+^ T on D0 and TNF on D8. In parallel, for absolute neutrophil counts on D8, 17 potential biomarkers were identified, the early ones being CCL2 and IFN-γ on D0, and CXCL8, CCL2, CXCL9, CXCL10 and IL-10 on D8. Regarding the absolute blast counts on D8, only IFN-γ also occurred on D15. For the absolute lymphocyte counts on D35, 7 early biomarkers were identified, CXCL9 also occurred on D0, and CD4^+^ T, IL-17A and IL-4 also occurred on D8. Finally, for platelet counts on D35, 19 potential early biomarkers were identified, CD8^+^ T, CCL5, TNF and IL-17A on D0, and Treg, CCL5 and TNF at D8.

## 4 Discussion

Leukemias have unique characteristics when compared to other neoplasms and solid tumors (32, 33). During malignant hematopoietic disorders such as B-ALL, intrinsic and extrinsic signals, including those participating in immunosurveillance, influence the pathway of cell differentiation and contribute to the prognosis and outcome of malignancy (34, 35). Advances in immunotherapeutic approaches have enabled better mapping of the immunological microenvironment of leukemia; however, the immunological background of B-ALL still needs further investigation (22, 36–38). Thus, we evaluated cell populations and the chemokine and cytokine network in children with B-ALL both on diagnosis and during treatment, with the aim of providing even more insights into the systemic immunological profile.

Our data on the cellular profile shows that, at the time of diagnosis (D0), pediatric patients with B-ALL showed a decrease in the frequency of NK, NKT and CD3^+^ T cells, which can be explained by the biological characteristics of the patients, who have severe cytopenia and poor reconstitution of the innate and adaptive immune response. Thus, although normal lymphoid and myeloid cells are present in the hematopoietic compartment, their hematopoietic progenitor cells are reduced in number and biological activity (39, 40). It is important to highlight that, in general, an increase in the frequency of cell populations was observed at the end of the induction therapy, indicating the recovery of normal hematopoiesis.

When we evaluated the subpopulations of CD3^+^ T cells, an increase in CD4^+^ T and CD8^+^ T cells was observed in relation to the children in the control group, suggesting immunosurveillance or the establishment of specific antitumor responses. In B-ALL, it has been proposed that tumor-specific T cells are not adequately activated; instead they are energized or exhausted (41, 42). T-cell dysfunction is modulated especially by Treg cells and results in decreased proliferative capacity and effector function, with increased expression of multiple inhibitory receptors, in order to contribute to immune tolerance (43, 44).

In fact, Treg cells have been shown to play a central role in the cellular immunosuppression of pediatric patients with B-ALL, either in the medullary compartment or in the peripheral blood. Some studies have examined whether the number of Treg CD4^+^ cells present in peripheral blood mononuclear cells (PBMC) is modulated in patients with B-ALL (45–48). Bhattacharya et al. reported that patients have a decrease in the frequency of CD4^+^CD25^+^ Treg cells compared to healthy children, but that, on the other hand, they co-express higher levels of FoxP3, IL-10, TGF-β and CD152, and have greater immunosuppressive activity (45). Other studies have reported increased CD4^+^CD25^+^FoxP3^+^ Treg cells, along with elevated levels of IL-10 and TGF-β (47–49).

Our findings are in line with the results of Bhattacharya et al. and demonstrate a tendency towards a decrease in the frequency of circulating CD4^+^CD25^+^FoxP3^+^ Treg cells at the time of diagnosis (D0), followed by a continuous increase during induction therapy (D8, D15 and D35) (**Figures 1A**, **2A**). Treg expansion in patients with B-ALL is expected, although these data may mean a migration of Treg CD4^+^CD25^+^FoxP3^+^ cells to the medullary compartment, the primary site of malignancy. Studies evaluating the bone marrow compartment of pediatric patients with B-ALL have reported an increase in the percentage of Treg cells compared to the peripheral blood systemic compartment and compared to the bone marrow of children without leukemia (48, 50).

The correlation between the frequency of tumor-infiltrating Treg cells and the prognosis has been described in several malignancies, including B-ALL (48, 50–52). Ultimately, these data indicate that, as in solid cancers, Treg cells infiltrate the medullary compartment and favor leukemic progression, thus determining a suppressive microenvironment. Reinforcing this scenario, although Treg cells showed a decreased frequency at D0, through the integrative networks, it was possible to observe that, specifically on D0, the patients presented a network that was characterized by the predominance of interactions mediated by circulatory Treg cells (**Figure 4**), as was also observed in a previous study in which we evaluated the bone marrow compartment of patients with B-ALL (16).

We also observed that, at the systemic level, these patients exhibit elevated levels of IL-10 on diagnosis. Concentrations decrease towards the end of remission induction therapy (**Figures 1B**, **2B**) as Treg cell-mediated correlations are lost (**Figure 4**). Alterations in the balance of anti-inflammatory/pro-inflammatory cytokines have already been described in ALL and B-ALL (37, 38, 41). Some studies indicate that leukemic cells hinder immune activation by creating an immunosuppressive microenvironment from the overproduction of anti-inflammatory cytokines and block the release of pro-inflammatory cytokines such as IFN-γ and TNF (53–55). Consistent with this equilibrium, by assessing the general signature of immunological mediators during induction therapy, we observed distinct production profiles throughout treatment. On D0, there was an attenuated production of immunological mediators, followed by a progressive increase on D8, D15 and D35, with remodulation towards a more pro-inflammatory profile (**Figure 3**).

In B-ALL, in addition to regulatory cytokines, leukemic cells express and benefit from the chemokine network to reduce immunogenicity, and promote leukemic growth and invasion (56, 57). Chemokines correspond to a class of cytokines that play key roles in inducing chemotaxis, differentiation and multiplication of leukocytes, which promotes tissue extravasation (58). In this context, the CXCL9, CXCL10 and CXCL11/CXCR3 axis has been an important focus for research. CXCL9-10-11 are selective ligands for CXCR3, which is preferentially expressed on the surface of monocytes, T cells, NK-cells, dendritic cells and cancer cells (59). These molecules also regulate the differentiation of naive T cells into T helper 1 (Th1) cells, in addition to conducting the migration of immune cells, such as CTLs, NK-cells, NKT and macrophages to their focal sites; therefore, this axis is considered essential to the command of the immune system (60). However, studies suggest that the axis may also play a tumorigenic role, increasing tumor proliferation and metastasis (61, 62).

To our knowledge, few studies have investigated the role of the CXCL9-10-11/CXCR3 axis in ALL (11, 36). Some studies have observed that a relapse of ALL patients was associated with the survival of leukemic cells in extramedullary tissues, such as the central nervous system (CNS), in which levels of antileukemic drugs are decreased (63). In T-ALL, CXCL10 levels in cerebrospinal fluid were significantly higher among patients with relapses in the CNS. Furthermore, treatment in a leukemic mouse model with the CXCR3 antagonist, AMG487, has been shown to significantly reduce leukemic CNS infiltration (11). In B-ALL, studies have found that IL-15 can up-regulate CXCR3 in B-cell precursors, stimulating the migration of leukemic cells towards the CXCL10 gradient, which is detectable in cerebrospinal fluid samples from patients with B-ALL (64). These results indicate that the CXCL10/CXCR3 axis can promote an important infiltration of ALL cells in the CNS and in other tissues, where chemotherapy levels are suboptimal.

In addition, CXCL10 released by monocytes promoted migration and invasive capacity of B-ALL CXCR3^+^ precursor cells by inducing MMP9 expression and activity, thus favoring metastatic dissemination (65). The CXCL10/CXCR3 axis has also been shown to contribute to recurrence of ALL, with an increase in the survival rate of leukemic cells during treatment. Mechanically, CXCL10 increased the viability and drug resistance of leukemic cells by stabilizing Bcl-2 and then inhibiting the activation of the caspase cascade induced by antineoplastic agents such as cytarabine, indicating that axis blockade may have a positive effect in reducing relapses in ALL patients (11). Finally, through the evaluation of the plasmatic levels of immunological mediators in the bone marrow of adult patients, it was observed that the levels of CXCL9 and CXCL10 are elevated on diagnosis, and were related to positivity for measurable residual disease (MRD) and to the stages of severity of the B-ALL (12).

We previously demonstrated that the network of soluble immunological mediators in the bone marrow can be an attractive source for investigation of potential prognostic biomarkers (17). However, it must be considered that the collection of bone marrow samples is a very invasive procedure, especially in pediatric populations. From another perspective, the identification of immunological elements that are predictive of therapeutic response at the systemic level, in peripheral blood as a liquid biopsy, allows sequential monitoring of the disease’s evolution. In this sense, we identified candidates for prognostic biomarkers, with excellent performance among the cell populations and systemic soluble immunological mediators that were evaluated (**Supplementary Tables 1**–**5** ). Five laboratory parameters for classifying the response to induction therapy were used as a basis: absolute neutrophil counts on D0; absolute neutrophil count on D0 and D8; blast count on D8; absolute lymphocyte count on D35; and platelet count on D35 (29–31). Incredibly, our data showed that several immunological mediators performed extremely well in classifying patients based on the parameters used, many of which performed very well as early biomarkers (D0 and D8), indicating the potential of these mediators as a liquid biopsy strategy (**Figure 5**).

Of note, the present study has some limitations. Because the FACSCalibur cytometer only permits the detection of four distinct fluorescence, it was not possible to expand the study panel to investigate the phenotype of some of the cell populations evaluated, such as CD4^+^ T and CD8^+^ T cells. Furthermore, the number of individuals ended up being reduced due to segment losses, however, it is important to consider the difficulty of working with pediatric populations and the barriers to carrying out longitudinal investigations. Additionally, some data’s were reported in MFI, in function the observed values were larger and provided more visually elegant graphs. It is important to highlight that, the values in MFI can provide estimates of the concentration of cytokines and chemokines present in the kits, as this value is used to calculate concentrations based on normalization calculations and standard curve calibration. Finally, we encourage additional studies in a paired way, evaluating the network of immunological mediators at the bone marrow and systemic level, and with the addition of functional assays, in order to allow an even more comprehensive view of the immunological profile and mechanisms in B-ALL.

## 5 Conclusion

**Figure 6:**
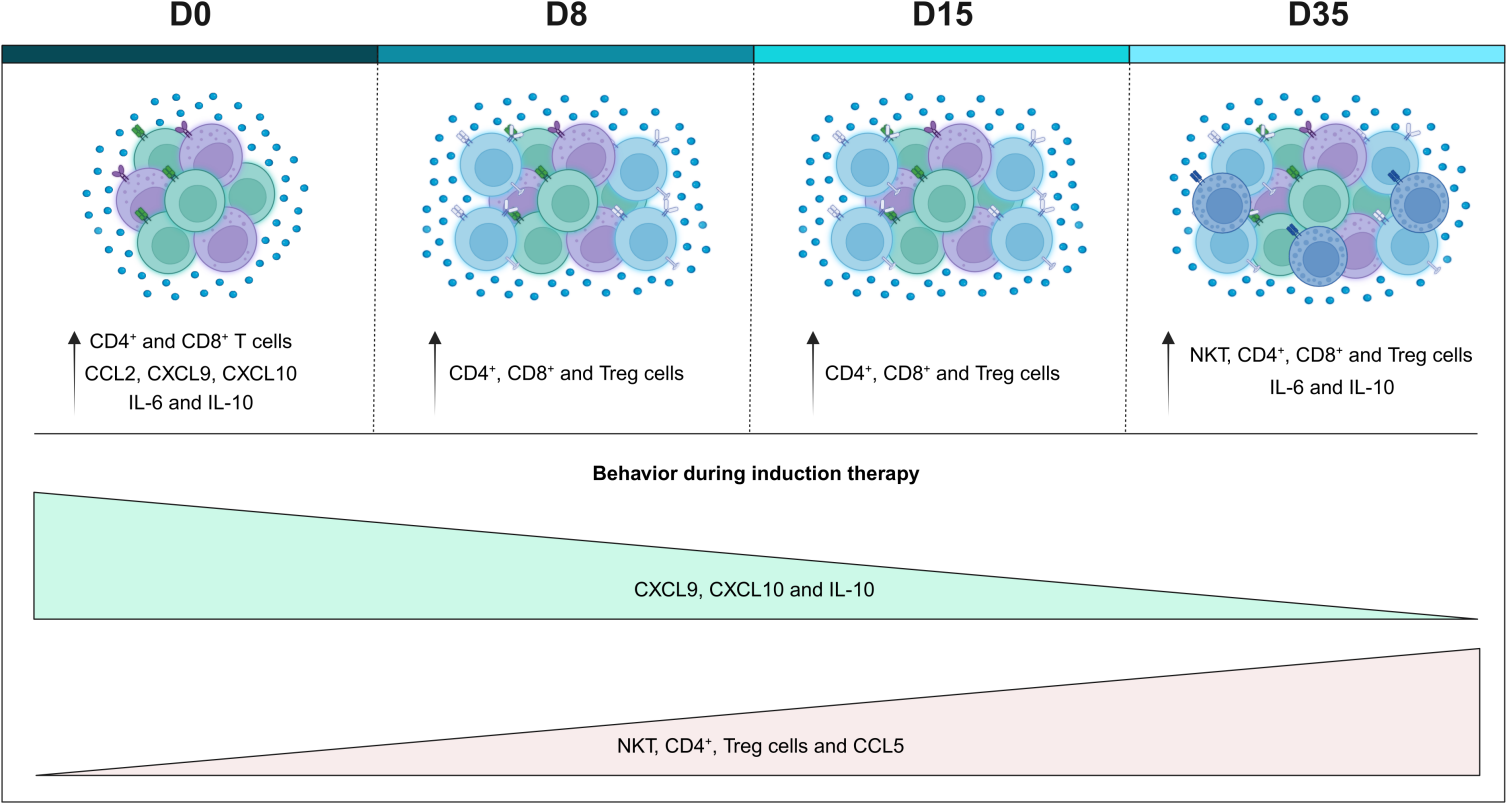
Remodeling of the systemic immunological profile of patients with B-ALL on the different days of induction therapy. Schematic presentation summarizing the main findings of the study, highlighting the immunological profile of B-ALL patients in relation to the controls, and the behavior of immunological mediators during remission induction therapy. Increased cell populations in B-ALL patients are represented by colors: (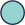) CD4^+^ T cells, (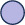) CD8^+^ T cells, Treg cells (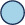) and (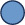) NKT-cells.

Finally, based on our results, we constructed a possible model of the remodeling of the systemic immunological profile during induction therapy (**Figure 6**). In summary, on D0, we generally observed a reduction in the frequency of cell populations, with the exception of CD4^+^ T and CD8^+^ T cells, which were increased, along with levels of CCL2, CXCL9, CXCL10, IL-6 and IL10, when compared to the CG. During the evaluation of the kinetics, it was possible to observe small changes on D8, such as a slight increase in the frequency of cell populations, and small changes in the levels of chemokines and cytokines, which were elevated. The same behavior was maintained on D15; while, on D35, it was possible to observe an opposite profile to that of D0, with an increase in NKT, CD3^+^ T, CD4^+^ T and Treg cells, together with CCL5, with a decline in CXCL9, CXCL10 and IL-10. Compared to the GC, there was an increase in NKT, CD3^+^ T, CD4^+^ T, CD8^+^ T and Treg cells, along with IL-6 and IL-10. Noteworthy, throughout the induction therapy, TNF levels remained low.

Furthermore, we provide important insights into the immune background in children with B-ALL both on diagnosis (D0) and during treatment (D8, D15 and D35). Our results demonstrate that patients with B-ALL present a complex and dynamic immune network during induction therapy. In addition, the frequency of cell populations and serum levels of chemokines and cytokines after remission induction still differ significantly when compared to the GC, indicating that this period is still short for complete immune reconstitution. Finally, we emphasize that the multiple immunological mediators show good performance in the classification of therapeutic response, many of which at an early stage, indicating their great potential as serum biomarkers.

## Data availability statement

The original contributions presented in the study are included in the article/**Supplementary Material**. Further inquiries can be directed to the corresponding author.

## Ethics statement

The studies involving humans were approved by Ethical Committee at Fundação Hospitalar de Hematologia e Hemoterapia do Amazonas (HEMOAM). The studies were conducted in accordance with the local legislation and institutional requirements. Written informed consent for participation in this study was provided by the participants’ legal guardians/next of kin.

## Author contributions

MC: Conceptualization, Investigation, Methodology, Writing – original draft, Formal Analysis, Writing – review and editing. FM-G: Formal Analysis, Investigation, Methodology, Writing – original draft, Writing – review and editing, Conceptualization, Project administration. BL: Methodology, Writing – original draft, Investigation. JN: Formal Analysis, Investigation, Methodology, Writing – original draft. NA: Investigation, Methodology, Writing – original draft, Project administration, Visualization. FS: Investigation, Methodology, Writing – original draft, Formal Analysis. CC: Methodology, Writing – original draft, Investigation, Data curation, Visualization. EA: Investigation, Methodology, Writing – original draft, Resources. JP: Investigation, Methodology, Writing – original draft, Validation. MB: Investigation, Methodology, Writing – original draft, Data curation. NF: Funding acquisition, Investigation, Methodology, Writing – original draft, Conceptualization. AT-C: Formal Analysis, Supervision, Writing – review and editing, Data curation, Validation. OM-F: Conceptualization, Data curation, Formal Analysis, Supervision, Validation, Writing – review and editing. AC: Conceptualization, Data curation, Formal Analysis, Funding acquisition, Methodology, Project administration, Supervision, Writing – original draft, Writing – review and editing. AM: Conceptualization, Funding acquisition, Supervision, Validation, Writing – review and editing.

## Supplementary material

Supplementary Figure 1 Representative flow cytometric analysis of cell populations. Peripheral blood lymphocytes were first selected based on their morphometric features (size/forward scatter - FSC and complexity/side scatter - SSC) on pseudo color plot **(A)**. Following, the phenotypic features were evaluated to quantify cell-subsets, including, within CD3^−^ and CD3^+^ events **(B)**, CD3^−^CD16^+^CD56^+^ NK-cells **(C)**, CD3^+^CD16^+^CD56^+^ NKT-cell **(D)**, CD8^+^ T-cells **(E)**, CD4^+^ T-cells **(F)** and CD4^+^CD25^+^FoxP3^+^ within CD3^+^ events-Treg cells **(G)**. All analysis were performed using the FlowJo software (version 9.4.1, TreeStar Inc. Ashland, OR, USA).

Supplementary Table 1 Performance of cell populations and soluble immunological mediators during induction therapy to classify B-ALL patients according to absolute neutrophil counts on diagnosis (D0).

Supplementary Table 2 Performance of cell populations and soluble immunological mediators during induction therapy to classify B-ALL patients according to absolute neutrophil counts during induction therapy (D8).

Supplementary Table 3 Performance of cell populations and soluble immunological mediators during induction therapy to classify B-ALL patients according to absolute blast counts during induction therapy (D8).

Supplementary Table 4 Performance of cell populations and soluble immunological mediators during induction therapy to classify B-ALL patients according to absolute lymphocyte counts after induction therapy (D35).

Supplementary Table 5 Performance of cell populations and soluble immunological mediators during induction therapy to classify B-ALL patients according to platelet counts after induction therapy (D35).
