# Supplementary figures and images for: Systemic immunological profile of children with B-cell acute lymphoblastic leukemia: performance of cell populations and soluble mediators as serum biomarkers
Source: Front Oncol. 2023 Dec 1;13:1290505. doi: 10.3389/fonc.2023.1290505 (PMC10722195; doi:10.3389/fonc.2023.1290505)

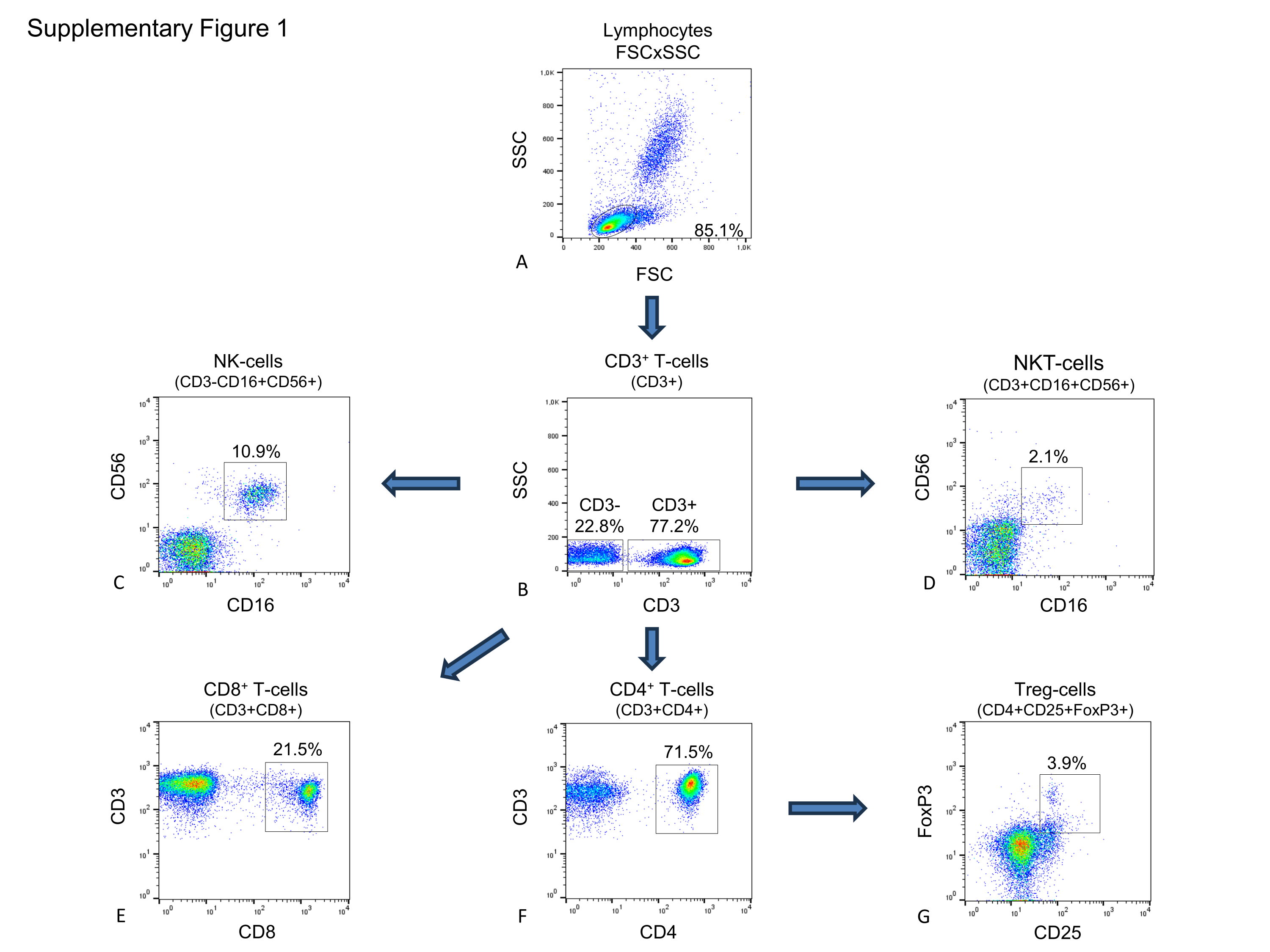

Supplement: Supplementary Figure 1 — Representative flow cytometric analysis of cell populations. Peripheral blood lymphocytes were first selected based on their morphometric features (size/forward scatter - FSC and complexity/side scatter - SSC) on pseudo color plot (A). Following, the phenotypic features were evaluated to quantify cell-subsets, including, within CD3− and CD3+ events (B), CD3−CD16+CD56+ NK-cells (C), CD3+CD16+CD56+ NKT-cell (D), CD8+ T-cells (E), CD4+ T-cells (F) and CD4+CD25+FoxP3+ within CD3+ events-Treg cells (G). All analysis were performed using the FlowJo software (version 9.4.1, TreeStar Inc. Ashland, OR, USA). [file Image_1.tiff]
