# Supplementary material for: Systemic immunological profile of children with B-cell acute lymphoblastic leukemia: performance of cell populations and soluble mediators as serum biomarkers
Source: Front Oncol. 2023 Dec 1;13:1290505. doi: 10.3389/fonc.2023.1290505 (PMC10722195; doi:10.3389/fonc.2023.1290505)
Supplement: Supplementary Table 1 — Performance of cell populations and soluble immunological mediators during induction therapy to classify B-ALL patients according to absolute neutrophil counts on diagnosis (D0). [file Table_1.docx]

**Supplementary Table 1**. Performance of cell populations and soluble immunological mediators during induction therapy to classify B-ALL patients according to absolute neutrophil counts on diagnosis (D0).

| Parameters | |  | Days of Induction Therapy | | | | | | | | | | | | | | |
| --- | --- | --- | --- | --- | --- | --- | --- | --- | --- | --- | --- | --- | --- | --- | --- | --- | --- |
|  |  |  | D0 | | |  | D8 | | |  | D15 | | |  | D35 | | |
|  |  |  | AUC (95% CI) |  | p |  | AUC (95% CI) |  | p |  | AUC (95% CI) |  | p |  | AUC (95% CI) |  | p |
|  |  |  |  |  |  |  |  |  |  |  |  |  |  |  |  |  |  |
| Cell Populations | NK |  | 0.71 (0.5-1.0) |  | 0.176 |  | 0.56 (0.3-0.9) |  | 0.711 |  | 0.62 (0.3-0.9) |  | 0.431 |  | 0.53 (0.2-0.8) |  | 0.861 |
|  | NKT |  | 0.67 (0.3-1.0) |  | 0.275 |  | 0.57 (0.3-0.8) |  | 0.643 |  | 0.66 (0.4-0.9) |  | 0.308 |  | 0.55 (0.2-0.9) |  | 0.760 |
|  | CD3^+^T |  | 0.53 (0.2-0.9) |  | 0.827 |  | 0.60 (0.3-0.9) |  | 0.516 |  | 0.60 (0.3-0.9) |  | 0.516 |  | 0.59 (0.2-1.0) |  | 0.570 |
|  | CD4^+^T |  | 0.63 (0.3-0.9) |  | 0.407 |  | 0.64 (0.3-0.9) |  | 0.354 |  | 0.51 (0.2-0.8) |  | 0.926 |  | 0.60 (0.3-0.9) |  | 0.512 |
|  | CD8^+^T |  | **0.77 (0.5-1.0)** |  | **0.050** |  | 0.53 (0.3-0.8) |  | 0.853 |  | 0.59 (0.3-0.9) |  | 0.547 |  | 0.53 (0.2-0.8) |  | 0.827 |
|  | Treg |  | 0.50 (0.1-0.9) |  | 0.999 |  | 0.57 (0.3-0.9) |  | 0.643 |  | 0.60 (0.3-0.9) |  | 0.516 |  | 0.56 (0.3-0.9) |  | 0.694 |
|  |  |  |  |  |  |  |  |  |  |  |  |  |  |  |  |  |  |
|  |  |  |  |  |  |  |  |  |  |  |  |  |  |  |  |  |  |
| Soluble Immunological Mediators | CXCL8 |  | 0.61 (0.2-1.0) |  | 0.458 |  | 0.64 (0.3-1.0) |  | 0.354 |  | 0.53 (0.2-0.9) |  | 0.827 |  | 0.66 (0.4-0.9) |  | 0.308 |
|  | CCL2 |  | 0.73 (0.4-1.0) |  | 0.126 |  | 0.67 (0.4-0.9) |  | 0.266 |  | 0.53 (0.3-0.8) |  | 0.827 |  | 0.73 (0.5-1.0) |  | 0.138 |
|  | CXCL9 |  | 0.59 (0.3-0.8) |  | 0.570 |  | 0.69 (0.4-0.9) |  | 0.228 |  | 0.51 (0.2-0.8) |  | 0.965 |  | 0.53 (0.2-0.8) |  | 0.853 |
|  | CCL5 |  | 0.63 (0.3-1.0) |  | 0.407 |  | 0.65 (0.3-1.0) |  | 0.331 |  | 0.63 (0.3-0.9) |  | 0.407 |  | 0.51 (0.2-0.8) |  | 0.926 |
|  | CXCL10 |  | 0.65 (0.4-0.9) |  | 0.315 |  | 0.67 (0.4-0.9) |  | 0.266 |  | 0.52 (0.2-0.9) |  | 0.895 |  | 0.58 (0.3-0.9) |  | 0.578 |
|  | IL-6 |  | 0.57 (0.3-0.9) |  | 0.662 |  | 0.57 (0.2-0.9) |  | 0.643 |  | 0.53 (0.2-0.9) |  | 0.861 |  | **0.77 (0.5-1.0)** |  | **0.050** |
|  | TNF |  | 0.67 (0.4-0.9) |  | 0.256 |  | **0.85 (0.7-1.0)** |  | **0.023** |  | 0.55 (0.3-0.8) |  | 0.760 |  | 0.54 (0.2-0.9) |  | 0.817 |
|  | IFN-γ |  | 0.62 (0.3-0.9) |  | 0.432 |  | 0.74 (0.5-1.0) |  | 0.115 |  | 0.65 (0.3-1.0) |  | 0.337 |  | 0.64 (0.3-0.9) |  | 0.379 |
|  | IL-17A |  | 0.53 (0.2-0.9) |  | 0.861 |  | 0.64 (0.4-0.9) |  | 0.379 |  | 0.57 (0.3-0.9) |  | 0.631 |  | 0.61 (0.3-1.0) |  | 0.487 |
|  | IL-4 |  | 0.52 (0.2-0.8) |  | 0.895 |  | 0.66 (0.3-1.0) |  | 0.287 |  | 0.59 (0.3-0.9) |  | 0.570 |  | 0.68 (0.4-0.9) |  | 0.247 |
|  | IL-10 |  | 0.59 (0.3-0.8) |  | 0.570 |  | 0.58 (0.3-0.9) |  | 0.610 |  | 0.64 (0.3-1.0) |  | 0.359 |  | 0.59 (0.3-0.9) |  | 0.578 |
|  | IL-2 |  | 0.57 (0.3-0.9) |  | 0.662 |  | 0.74 (0.5-1.0) |  | 0.115 |  | **0.80 (0.6-1.0)** |  | **0.049** |  | **0.84 (0.6-1.0)** |  | **0.029** |
|  |  |  |  |  |  |  |  |  |  |  |  |  |  |  |  |  |  |

B-ALL = B-cell acute lymphoblastic leukemia (n=20) was classified according to absolute neutrophil counts (ANC) om diagnosis (D0); ANC > 1x10^3^ cells/mm^3^ was considered a putative laboratory marker for better disease outcome. AUC = area under the receiver operating characteristic (ROC) curve; CI = confidence interval; Significance was considered when p was <0.05.
