# Supplementary material for: Systemic immunological profile of children with B-cell acute lymphoblastic leukemia: performance of cell populations and soluble mediators as serum biomarkers
Source: Front Oncol. 2023 Dec 1;13:1290505. doi: 10.3389/fonc.2023.1290505 (PMC10722195; doi:10.3389/fonc.2023.1290505)
Supplement: Supplementary Table 2 — Performance of cell populations and soluble immunological mediators during induction therapy to classify B-ALL patients according to absolute neutrophil counts during induction therapy (D8). [file Table_2.docx]

**Supplementary Table 2.** Performance of cell populations and soluble immunological mediators during induction therapy to classify B-ALL patients according to absolute neutrophil counts during induction therapy (D8).

| **Parameters** | |  | **Days of Induction Therapy** | | | | | | | | | | | | | | |
| --- | --- | --- | --- | --- | --- | --- | --- | --- | --- | --- | --- | --- | --- | --- | --- | --- | --- |
|  |  |  | **D0** | | |  | **D8** | | |  | **D15** | | |  | **D35** | | |
|  |  |  | **AUC (95% CI)** |  | **p** |  | **AUC (95% CI)** |  | **p** |  | **AUC (95% CI)** |  | **p** |  | **AUC (95% CI)** |  | **p** |
|  |  |  |  |  |  |  |  |  |  |  |  |  |  |  |  |  |  |
| **Cell Populations** | NK |  | 0.78 (0.5-1.0) |  | 0.207 |  | 0.68 (0.3-1.0) |  | 0.425 |  | 0.57 (0.3-0.8) |  | 0.739 |  | 0.56 (0.3-0.8) |  | 0.801 |
|  | NKT |  | 0.83 (0.7-1.0) |  | 0.130 |  | 0.62 (0.4-0.8) |  | 0.595 |  | 0.53 (0.3-0.8) |  | 0.894 |  | 0.56 (0.0-1.0) |  | 0.801 |
|  | CD3^+^T |  | 0.75 (0.5-1.0) |  | 0.256 |  | 0.53 (0.3-0.8) |  | 0.894 |  | 0.74 (0.4-1.0) |  | 0.287 |  | 0.75 (0.6-0.9) |  | 0.256 |
|  | CD4^+^T |  | 0.61 (0.4-0.8) |  | 0.614 |  | 0.59 (0.4-0.8) |  | 0.690 |  | 0.59 (0.3-0.8) |  | 0.690 |  | 0.64 (0.4-0.9) |  | 0.528 |
|  | CD8^+^T |  | 0.56 (0.2-0.9) |  | 0.801 |  | 0.53 (0.2-0.8) |  | 0.894 |  | 0.59 (0.4-0.8) |  | 0.690 |  | 0.64 (0.4-0.9) |  | 0.528 |
|  | Treg |  | 0.85 (0.5-1.0) |  | 0.175 |  | 0.53 (0.2-0.8) |  | 0.894 |  | **0.97 (0.9-1.0)** |  | **0.033** |  | 0.56 (0.2-0.9) |  | 0.801 |
|  |  |  |  |  |  |  |  |  |  |  |  |  |  |  |  |  |  |
|  |  |  |  |  |  |  |  |  |  |  |  |  |  |  |  |  |  |
| **Soluble Immunological Mediators** | CXCL8 |  | 0.83 (0.6-1.0) |  | 0.130 |  | **0.97 (0.9-1.0)** |  | **0.033** |  | **1.00 (1.0-1.0)** |  | **0.023** |  | **1.00 (1.0-1.0)** |  | **0.023** |
|  | CCL2 |  | **0.92 (0.8-1.0)** |  | **0.050** |  | **0.94 (0.8-1.0)** |  | **0.046** |  | **0.94 (0.8-1.0)** |  | **0.043** |  | 0.68 (0.3-1.0) |  | 0.425 |
|  | CXCL9 |  | 0.61 (0.4-0.9) |  | 0.614 |  | **1.00 (1.0-1.0)** |  | **0.023** |  | **1.00 (1.0-1.0)** |  | **0.023** |  | 0.68 (0.4-1.0) |  | 0.425 |
|  | CCL5 |  | 0.86 (0.7-1.0) |  | 0.101 |  | 0.68 (0.2-1.0) |  | 0.425 |  | 0.53 (0.3-0.8) |  | 0.899 |  | 0.56 (0.3-0.8) |  | 0.790 |
|  | CXCL10 |  | 0.56 (0.3-0.8) |  | 0.801 |  | **0.97 (0.9-1.0)** |  | **0.033** |  | **0.97 (0.9-1.0)** |  | **0.032** |  | **0.91 (0.8-1.0)** |  | **0.050** |
|  | IL-6 |  | 0.51 (0.2-0.8) |  | 0.949 |  | 0.79 (0.6-1.0) |  | 0.184 |  | 0.74 (0.5-1.0) |  | 0.284 |  | 0.82 (0.6-1.0) |  | 0.143 |
|  | TNF |  | 0.69 (0.5-0.9) |  | 0.377 |  | 0.69 (0.3-1.0) |  | 0.387 |  | **0.92 (0.8-1.0)** |  | **0.050** |  | 0.60 (0.2-1.0) |  | 0.698 |
|  | IFN-γ |  | **0.93 (0.8-1.0)** |  | **0.050** |  | 0.51 (0.2-0.8) |  | 0.947 |  | 0.64 (0.1-1.0) |  | 0.528 |  | **0.90 (0.7-1.0)** |  | **0.050** |
|  | IL-17A |  | 0.50 (0.0-1.0) |  | 0.999 |  | 0.53 (0.1-1.0) |  | 0.894 |  | 0.71 (0.3-1.0) |  | 0.344 |  | 0.78 (0.6-1.0) |  | 0.206 |
|  | IL-4 |  | 0.68 (0.3-1.0) |  | 0.412 |  | 0.72 (0.3-1.0) |  | 0.319 |  | 0.82 (0.5-1.0) |  | 0.147 |  | 0.60 (0.2-1.0) |  | 0.642 |
|  | IL-10 |  | 0.53 (0.2-0.8) |  | 0.899 |  | **0.94 (0.8-1.0)** |  | **0.046** |  | **0.90 (0.8-1.0)** |  | **0.050** |  | 0.79 (0.5-1.0) |  | 0.184 |
|  | IL-2 |  | 0.51 (0.1-1.0) |  | 0.949 |  | 0.78 (0.4-1.0) |  | 0.206 |  | 0.55 (0.0-1.0) |  | 0.846 |  | 0.65 (0.4-0.9) |  | 0.506 |
|  |  |  |  |  |  |  |  |  |  |  |  |  |  |  |  |  |  |

B-ALL = B-cell acute lymphoblastic leukemia (n=20) was classified according to absolute neutrophil counts (ANC) during induction therapy (D8); ANC > 1x10^3^ cells/mm^3^ was considered a putative laboratory marker for better disease outcome. AUC = area under the receiver operating characteristic (ROC) curve; CI = confidence interval; Significance was considered when p was <0.05.
