# Supplementary material for: Systemic immunological profile of children with B-cell acute lymphoblastic leukemia: performance of cell populations and soluble mediators as serum biomarkers
Source: Front Oncol. 2023 Dec 1;13:1290505. doi: 10.3389/fonc.2023.1290505 (PMC10722195; doi:10.3389/fonc.2023.1290505)
Supplement: Supplementary Table 3 — Performance of cell populations and soluble immunological mediators during induction therapy to classify B-ALL patients according to absolute blast counts during induction therapy (D8). [file Table_3.docx]

**Supplementary Table 3.** Performance of cell populations and soluble immunological mediators during induction therapy to classify B-ALL patients according to absolute blast counts during induction therapy (D8).

| **Parameters** | |  | **Days of Induction Therapy** | | | | | | | | | | | | | | |
| --- | --- | --- | --- | --- | --- | --- | --- | --- | --- | --- | --- | --- | --- | --- | --- | --- | --- |
|  |  |  | **D0** | | |  | **D8** | | |  | **D15** | | |  | **D35** | | |
|  |  |  | **AUC (95% CI)** |  | **p** |  | **AUC (95% CI)** |  | **p** |  | **AUC (95% CI)** |  | **p** |  | **AUC (95% CI)** |  | **p** |
|  |  |  |  |  |  |  |  |  |  |  |  |  |  |  |  |  |  |
| **Cell Populations** | NK |  | 0.62 (0.4-0.9) |  | 0.409 |  | 0.63 (0.3-0.9) |  | 0.380 |  | 0.56 (0.3-0.8) |  | 0.677 |  | 0.56 (0.3-0.9) |  | 0.680 |
|  | NKT |  | 0.51 (0.2-0.8) |  | 0.934 |  | 0.59 (0.3-0.9) |  | 0.539 |  | 0.71 (0.5-0.9) |  | 0.164 |  | 0.54 (0.2-0.9) |  | 0.804 |
|  | CD3^+^T |  | 0.70 (0.5-0.9) |  | 0.173 |  | 0.65 (0.4-0.9) |  | 0.292 |  | 0.56 (0.3-0.8) |  | 0.711 |  | 0.71 (0.5-1.0) |  | 0.148 |
|  | CD4^+^T |  | 0.63 (0.4-0.9) |  | 0.364 |  | 0.53 (0.3-0.8) |  | 0.860 |  | 0.53 (0.3-0.8) |  | 0.853 |  | 0.64 (0.4-0.9) |  | 0.322 |
|  | CD8^+^T |  | 0.52 (0.2-0.8) |  | 0.901 |  | 0.51 (0.2-0.8) |  | 0.965 |  | 0.58 (0.3-0.8) |  | 0.610 |  | 0.71 (0.5-0.9) |  | 0.137 |
|  | Treg |  | 0.56 (0.3-0.8) |  | 0.680 |  | 0.65 (0.4-0.9) |  | 0.292 |  | 0.57 (0.2-0.9) |  | 0.643 |  | 0.60 (0.3-0.9) |  | 0.509 |
|  |  |  |  |  |  |  |  |  |  |  |  |  |  |  |  |  |  |
|  |  |  |  |  |  |  |  |  |  |  |  |  |  |  |  |  |  |
| **Soluble Immunological Mediators** | CXCL8 |  | 0.57 (0.3-0.9) |  | 0.620 |  | 0.51 (0.2-0.8) |  | 0.930 |  | 0.58 (0.3-0.9) |  | 0.563 |  | 0.59 (0.3-0.9) |  | 0.578 |
|  | CCL2 |  | 0.57 (0.3-0.8) |  | 0.620 |  | 0.62 (0.3-0.9) |  | 0.429 |  | 0.60 (0.3-0.9) |  | 0.509 |  | 0.50 (0.2-0.8) |  | 0.999 |
|  | CXCL9 |  | 0.55 (0.2-0.8) |  | 0.741 |  | 0.64 (0.4-0.9) |  | 0.334 |  | 0.52 (0.3-0.8) |  | 0.869 |  | 0.69 (0.3-1.0) |  | 0.228 |
|  | CCL5 |  | 0.54 (0.2-0.8) |  | 0.804 |  | 0.51 (0.2-0.8) |  | 0.930 |  | 0.60 (0.3-0.9) |  | 0.509 |  | 0.64 (0.3-0.9) |  | 0.379 |
|  | CXCL10 |  | 0.54 (0.3-0.8) |  | 0.804 |  | 0.71 (0.5-1.0) |  | 0.160 |  | 0.50 (0.2-0.8) |  | 0.999 |  | 0.66 (0.4-0.9) |  | 0.308 |
|  | IL-6 |  | 0.58 (0.3-0.9) |  | 0.563 |  | 0.53 (0.2-0.8) |  | 0.826 |  | 0.63 (0.4-0.9) |  | 0.364 |  | 0.64 (0.4-0.9) |  | 0.354 |
|  | TNF |  | 0.52 (0.2-0.8) |  | 0.869 |  | 0.52 (0.2-0.8) |  | 0.895 |  | 0.53 (0.2-0.8) |  | 0.836 |  | 0.68 (0.4-1.0) |  | 0.247 |
|  | IFN-γ |  | 0.60 (0.3-0.9) |  | 0.509 |  | 0.58 (0.3-0.9) |  | 0.598 |  | **0.96 (0.9-1.0)** |  | **0.001** |  | 0.62 (0.4-0.9) |  | 0.431 |
|  | IL-17A |  | 0.62 (0.3-0.9) |  | 0.409 |  | 0.53 (0.3-0.8) |  | 0.826 |  | 0.57 (0.3-0.9) |  | 0.620 |  | 0.59 (0.3-0.9) |  | 0.547 |
|  | IL-4 |  | 0.67 (0.4-0.9) |  | 0.248 |  | 0.58 (0.2-0.9) |  | 0.598 |  | 0.70 (0.4-1.0) |  | 0.173 |  | 0.61 (0.3-0.9) |  | 0.487 |
|  | IL-10 |  | 0.64 (0.4-0.9) |  | 0.322 |  | 0.55 (0.2-0.9) |  | 0.758 |  | 0.68 (0.4-0.9) |  | 0.216 |  | 0.61 (0.3-0.9) |  | 0.487 |
|  | IL-2 |  | 0.52 (0.2-0.8) |  | 0.869 |  | 0.56 (0.2-0.9) |  | 0.661 |  | 0.63 (0.3-0.9) |  | 0.364 |  | 0.54 (0.2-0.8) |  | 0.817 |
|  |  |  |  |  |  |  |  |  |  |  |  |  |  |  |  |  |  |

B-ALL = B-cell acute lymphoblastic leukemia (n=20) was classified according to absolute blast counts (ABC) during induction therapy (D8); Undetectable ABC at D8 was considered a putative laboratory marker for better disease outcome. AUC = area under the receiver operating characteristic (ROC) curve; CI = confidence interval; Significance was considered when p was <0.05.
