# Supplementary material for: Systemic immunological profile of children with B-cell acute lymphoblastic leukemia: performance of cell populations and soluble mediators as serum biomarkers
Source: Front Oncol. 2023 Dec 1;13:1290505. doi: 10.3389/fonc.2023.1290505 (PMC10722195; doi:10.3389/fonc.2023.1290505)
Supplement: Supplementary Table 4 — Performance of cell populations and soluble immunological mediators during induction therapy to classify B-ALL patients according to absolute lymphocyte counts after induction therapy (D35). [file Table_4.docx]

**Supplementary Table 4.** Performance of cell populations and soluble immunological mediators during induction therapy to classify B-ALL patients according to absolute lymphocyte counts after induction therapy (D35).

| **Parameters** | |  | **Days of Induction Therapy** | | | | | | | | | | | | | | |
| --- | --- | --- | --- | --- | --- | --- | --- | --- | --- | --- | --- | --- | --- | --- | --- | --- | --- |
|  |  |  | **D0** | | |  | **D8** | | |  | **D15** | | |  | **D35** | | |
|  |  |  | **AUC (95% CI)** |  | **p** |  | **AUC (95% CI)** |  | **p** |  | **AUC (95% CI)** |  | **p** |  | **AUC (95% CI)** |  | **p** |
|  |  |  |  |  |  |  |  |  |  |  |  |  |  |  |  |  |  |
| **Cell Populations** | NK |  | 0.58 (0.3-0.9) |  | 0.568 |  | 0.69 (0.4-0.9) |  | 0.165 |  | 0.53 (0.3-0.8) |  | 0.836 |  | 0.58 (0.3-0.9) |  | 0.543 |
|  | NKT |  | 0.59 (0.3-0.8) |  | 0.518 |  | 0.57 (0.3-0.8) |  | 0.624 |  | 0.56 (0.3-0.8) |  | 0.679 |  | 0.63 (0.3-0.9) |  | 0.342 |
|  | CD3^+^T |  | 0.51 (0.2-0.8) |  | 0.939 |  | 0.52 (0.3-0.8) |  | 0.870 |  | 0.58 (0.3-0.9) |  | 0.563 |  | 0.56 (0.3-0.8) |  | 0.648 |
|  | CD4^+^T |  | 0.61 (0.4-0.9) |  | 0.403 |  | **0.82 (0.6-1.0)** |  | **0.017** |  | 0.51 (0.2-0.8) |  | 0.969 |  | 0.61 (0.3-0.9) |  | 0.409 |
|  | CD8^+^T |  | 0.69 (0.4-0.9) |  | 0.148 |  | 0.56 (0.3-0.8) |  | 0.653 |  | 0.59 (0.3-0.9) |  | 0.508 |  | 0.67 (0.4-0.9) |  | 0.210 |
|  | Treg |  | 0.56 (0.3-0.8) |  | 0.676 |  | 0.54 (0.3-0.8) |  | 0.744 |  | **0.84 (0.7-1.0)** |  | **0.013** |  | 0.53 (0.2-0.8) |  | 0.849 |
|  |  |  |  |  |  |  |  |  |  |  |  |  |  |  |  |  |  |
|  |  |  |  |  |  |  |  |  |  |  |  |  |  |  |  |  |  |
| **Soluble Immunological Mediators** | CXCL8 |  | 0.52 (0.2-0.8) |  | 0.909 |  | 0.50 (0.2-0.8) |  | 0.999 |  | 0.57 (0.3-0.8) |  | 0.621 |  | **0.82 (0.6-1.0)** |  | **0.020** |
|  | CCL2 |  | 0.51 (0.2-0.8) |  | 0.969 |  | 0.66 (0.4-0.9) |  | 0.253 |  | 0.62 (0.3-0.9) |  | 0.382 |  | 0.64 (0.4-0.9) |  | 0.321 |
|  | CXCL9 |  | **0.78 (0.6-1.0)** |  | **0.036** |  | 0.54 (0.3-0.8) |  | 0.744 |  | 0.70 (0.5-0.9) |  | 0.138 |  | 0.56 (0.3-0.8) |  | 0.679 |
|  | CCL5 |  | 0.55 (0.3-0.8) |  | 0.732 |  | 0.55 (0.3-0.8) |  | 0.713 |  | 0.56 (0.3-0.8) |  | 0.676 |  | 0.65 (0.4-0.9) |  | 0.283 |
|  | CXCL10 |  | 0.62 (0.4-0.9) |  | 0.382 |  | 0.64 (0.4-0.9) |  | 0.288 |  | 0.54 (0.3-0.8) |  | 0.790 |  | 0.57 (0.3-0.9) |  | 0.620 |
|  | IL-6 |  | 0.54 (0.3-0.8) |  | 0.790 |  | 0.59 (0.3-0.9) |  | 0.513 |  | 0.57 (0.3-0.8) |  | 0.594 |  | 0.50 (0.2-0.8) |  | 0.999 |
|  | TNF |  | 0.67 (0.4-0.9) |  | 0.196 |  | 0.53 (0.3-0.8) |  | 0.860 |  | **0.78 (0.6-1.0)** |  | **0.033** |  | 0.53 (0.3-0.8) |  | 0.804 |
|  | IFN-γ |  | 0.55 (0.3-0.8) |  | 0.704 |  | 0.68 (0.4-0.9) |  | 0.177 |  | 0.53 (0.3-0.8) |  | 0.790 |  | 0.67 (0.4-0.9) |  | 0.215 |
|  | IL-17A |  | 0.50 (0.2-0.8) |  | 0.999 |  | **0.87 (0.7-1.0)** |  | **0.006** |  | 0.61 (0.4-0.9) |  | 0.425 |  | 0.54 (0.2-0.8) |  | 0.772 |
|  | IL-4 |  | 0.58 (0.3-0.8) |  | 0.543 |  | **0.74 (0.5-1.0)** |  | **0.050** |  | 0.53 (0.3-0.8) |  | 0.819 |  | 0.59 (0.3-0.9) |  | 0.535 |
|  | IL-10 |  | 0.63 (0.4-0.9) |  | 0.342 |  | 0.53 (0.3-0.8) |  | 0.838 |  | 0.58 (0.3-0.9) |  | 0.568 |  | 0.70 (0.5-0.9) |  | 0.137 |
|  | IL-2 |  | 0.57 (0.3-0.8) |  | 0.621 |  | 0.69 (0.4-0.9) |  | 0.165 |  | 0.59 (0.3-0.8) |  | 0.518 |  | 0.70 (0.5-0.9) |  | 0.148 |
|  |  |  |  |  |  |  |  |  |  |  |  |  |  |  |  |  |  |

B-ALL = B-cell acute lymphoblastic leukemia (n=20) was classified according to absolute lymphocyte counts (ALC) after induction therapy (D35); ALC > 1.5 x 10^3^ cells/mm^3^ at D35 was considered a putative laboratory marker for better disease outcome. AUC = area under the receiver operating characteristic (ROC) curve; CI = confidence interval; Significance was considered when p was <0.05.
